# Supplementary figures and images for: Understanding the response in Pugionium cornutum (L.) Gaertn. seedling leaves under drought stress using transcriptome and proteome integrated analysis
Source: PeerJ. 2023 Apr 3;11:e15165. doi: 10.7717/peerj.15165 (PMC10078451; doi:10.7717/peerj.15165)

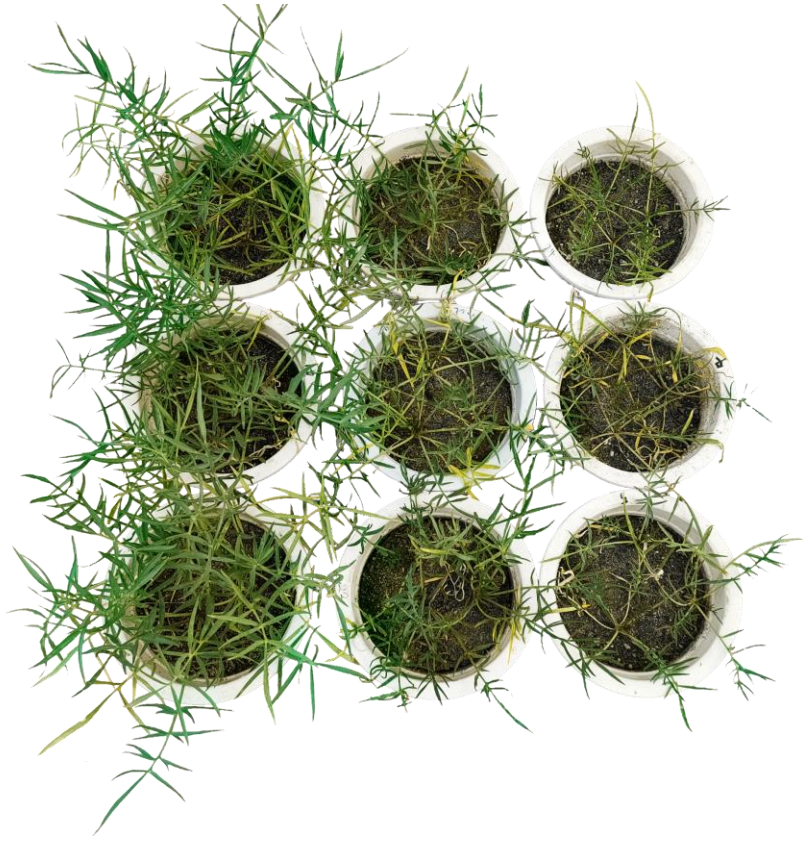

Supplement: Supplemental Information 7 [file peerj-11-15165-s007.pdf]
